# Gastric Carcinoma2Read before the Roswell Park Medical Club, May 9, 1898.

**Published:** 1898-08

**Authors:** Julius Ullman

**Affiliations:** Buffalo, N. Y.; Instructor in clinical medicine, Medical Department, University of Buffalo: attending physician at the German Hospital Dispensary; 400 Franklin St.


### GASTRIC CARCINOMA.[2]

### THE PRESENCE OF THE FADEN (OPPLER-BOAS) BACILLUS AS AN IMPORTANT DIAGNOSTIC FACTOR IN GASTRIC CARCINOMA.

By JULIUS ULLMAN, M. D., Buffalo, N. Y.
Instructor in clinical medicine, Medical Department, University of Buffalo: attending physician at the German Hospital Dispensary.

IN NO department of internal medicine has there been more marked progress during the past two decades than in the domain of gastric diseases. The success lies in the introduction of the stomach pump by Kussmaul and the stomach-sound by Leube, and following these the use of the soft stomach-tube by Oser and Ewald, so that by their use we were first enabled to study the gastric contents. This stimulated investigations in a branch of medicine which up to that time was little known. The name dyspepsia, that harbinger of ignorance,

1. Transactions of the Medical Society of the State of New York, 1898.
2. Read before the Roswell Park Medical Club, May 9, 1898.

is being replaced by names of diseases whose pathology is known and therefore which can be relieved by treatment other than empirical. By a direct examination of stomach contents we are now enabled to conveniently classify the diseases of the stomach.

It is not my purpose to speak of the value of chemical analyses, but briefly to allude to the benefits of microscopical examinations in the diagnosis of diseases of the stomach, and more especially in gastric carcinoma. Although this method of examination may not be an absolute determining factor of the underlying morbid pathological anatomical conditions, still in conjunction with other subjective and objective symptoms it is of absolute importance and often leads to a certainty where otherwise there would be doubt.

Limiting ourselves to gastric carcinoma, it has been established from examination of the blood that there is a leucocytosis, especially in such cases where the neoplasm has undergone ulceration or necrosis or where there are metastases in other organs (Clinical examination of blood—Cabot). Many observers, among whom may be mentioned Hartung (Wiener Klinische Woch., p. 697, 1895) and Schneyer, (Zeit. für Inner. Med.) have found the absence of the ordinary leucocytosis of digestion in gastric carcinoma.

We are much indebted to Boas and Paul Cohenheim (Archives für Verdaungs Krankheiten, Bd., p. 274) for calling to our attention the importance of the microscopical examination of particles of mucosa obtained from the washings of the stomach in gastric cancer and other diseases.

I have chosen as a subject one, which to me at least for the past two or three years, has been of interest—namely, the presence of Faden (Oppler-Boas) bacillus as an important diagnostic factor in gastric carcinoma. This organism has invariably been found by me to be present in suspected cases of gastric carcinoma, in a number of which the diagnosis was later verified by the increasing symptoms of the progress of the disease and, of gıeater importance, by the microscopical detection of the tumor post monem.

This organism was first detected in the stomach contents by B. Oppler (Deutsche Med. Woch., 1895, No. 5) while an assistant at the Poliklinik of Boas, and in the same year was described by Schlesinger and Kaufmann, (Wiener Klinische Rundschau, 1895,) all of whom assert that a large number of these bacilli found in the stomach contents is indicative of gastric carcinoma.

When we consider the chance for recovery or prolongation of life by operative interference, which is given to a patient if the disease may be diagnosticated early enough, and the fact that this organism may be detected when there still is no tumor palpable and when the cachexia may as yet not be sufficiently advanced to make an operation impracticable, you will agree with me that we have in its presence a sign of great and practical importance.

As described by Oppler, these bacilli are long, slender and non-motile and may be seen in every field, lying between the cellular elements and particles of food of stomach contents.

Strauss and Bialcaom (Zeit. für Klin. Med., XXVIII., No. 5 and 6,) have been able to get cultures of them. They do not grow on gelatine or agar, but on a mixture of agar two parts and one part of filtered concentrated glucose and carcinomatous juice.

Kaufmann and Schlesinger obtained growths on meat peptone agar and glucose agar. Their presence seems to be favored by an absence of free hydrochloric acid and the presence of lactic acid. Kaufmann and others assert that these organisms have the power of forming lactic acid from various kinds of sugar. In staining them you proceed as with other microörganisms, spread them with the stomach contents on a cover glass, dry, fix by passing through the flame and stain with any of the aniline dyes (methylene blue, gentian violet and the like). They also take on the Gram stain. They may be seen with the high power and better still with the oil immersion.

If you ask me what pathological relationship exists between the neoplasm and the presence of the bacteria, I must admit that in all probability they are a result of the carcinoma and not its cause, for if we consider the great loss of motility and the ensuing dilatation with its food stagnation and putrefaction, due to the absence of free hydrochloric acid ; here may be found a cause for their presence.

Rosenheim and Richter (Zeit. für Klin. Med., Bd. XXVIII., Nos. 5 and 6) claim to have seen these organisms in other than carcinomatous cases, but so far as my observations and others whom I have mentioned in this paper are concerned, they are present only in carcinoma and never in other structural or functional gastric diseases. Their presence is, therefore, not only of value in the diagnosis of gastric carcinoma, but in their absence we may exclude, in conjunction with their symptoms, other diseased conditions which may at sometime in their course very much simulate gastric carcinoma.

In gastrectasia, due to a benign obstruction, there are microörganisms called the raccinæ ventriculi present. They are observed in biscuit or bale-shaped groups of four, eight or sixteen, arranged together. During the progress of a gastrectasia they may be replaced by the Oppler-Boas bacilli and this indicates that a pyloric obstruction, which may have been adenoma or a cicatrix of an old chronic ulcer has become an adeno-carcinoma and the seat of the ulcer undergone a carcinomatous infiltration. There are other diseases, such as gastritis anacida and achylia gastrica where, as is common in carcinoma, free hydrochloric acid is absent, in which I have been unable to see these organism.

I will briefly allude to the experience of observers on these organism. Kaufmann has found the organisms nineteen times in a series of twenty cases. Oppler does not mention the number of cases in which he has detected them, but from my experience at the Boas Poliklinik, where Oppler made his first observations, he must have seen a great many cases in which they were present, for I was able to detect them in this large clinic in at least eight or ten cases, which were diagnosticated as gastric carcinoma.

Boas in his book (Allgemeine Diagnostik und Therapie der Magenkrankheiten, Dr. I. Boas, Theil II., page 182,) speaks of the significance of these organisms.

In 1896 I found them present in a case of Dr. Allen Jones, of this city. He reported this case in his excellent paper on gastric carcinoma (Med. News, 1896). In this patient there was no palpable tumor or perceptible dilatation. Dr. Jones recently told me that this case died. There developed a palpable tumor; blood and fragments of ulcerating growth were frequently found in the washings from the stomach afterwards.

Hemmeter (Diseases of the stomach, p. 529)says that these organisms are a very important diagnostic sign in gastric cancer. In sixteen cases thus far examined by him he found it present in fourteen.

Charles G. Stockton and Allen Jones in their article on carcinoma (Am. System of Prac.) also attach importance to their presence. They say "the Faden-like bacillus is often present in carcinoma, but has not been found in other diseases."

Prof. Reigel, of Giesen, (Die Erkrankungen des Magens, p. 74, 1896,) says "the fact of the constant and enormous presence of these bacilli in cancer, I can substantiate from personal observation. If they are not pathognomonic they are very useful from a point of diagnosis."

I herewith report a number of cases in which I have found them present, all of which were found to be carcinoma on post mortem examination.

CASE I.—W. A., aged 48.—In the practice of Dr. A. T. Lytle.—Policeman; hard drinker. Since 1895 complained of gastric distress after eating and weakness. Was in General Hospital in 1895. Vomiting increased as did weakness, also a pernicious anemia. Red corpuscles, 1,209,375; white corpuscles, 12,500; hemoglobin, 25 per cent. All of the gastric symptoms increased and there was no great emaciation. There was absence of free hydrochloric acid, the presence of lactic acid and the Faden bacilli in large numbers. Died, October 30, 1896.

Post-mortem. Pancreas enlarged; nodules in the liver; a gastric carcinoma located on the seat of an old gastric ulcer, on the posterior wall of the stomach, between the pylorus and cardia. In this case there was no marked emaciation; because of the location of the neoplasm there was no pyloric obstruction.

CASE II.—B. B., aged 49, infantryman, seen with Major Appel at the Army hospital in 1895. History of excessive drinking for a number of years. At time of examination the patient was much emaciated, cachectic, very anemic and vomited excessively. There was also marked tenderness and pain over the whole abdominal area. Examination of blood showed a marked leucocytosis and a chloroanemia. Patient died, and the necropsy performed by Dr. H. U. Williams revealed an ulcerating carcinoma on the posterior wall of the stomach near the pylorus. There were nodules in the liver and the lodes were also involved. Microscopical examination of the tumor proved it to be an encephaloid carcinoma.

CASE III.—A post mortem of a case in the practice of Dr. Gustav Pohl, in which I was enabled to get some of the stomach contents and in which the bacilli were present in large quantities, the tumor was situated at the pylorus and was ulcerative.

CASE IV.—A case at the General Hospital in which it, together with other well marked-symptoms of advanced carcinoma were present. The patient left the hospital, but I have since learned of his decease.

Dr. H. J. Knickerbocker, under my supervision, collected four cases, in all of which he found the organisms present.

Lastly I have to relate a case where its absence greatly aided in the diagnosis of a benign cicatricial contraction at the pylorus.

CASE V.—J. G., aged 59, cooper, German, seen at General Hospital. For six or seven years had feeling of distress after eating. Vomits and vomitus has sour taste and disagreeable odor. Lost greatly in weight and strength, to such a degree that he could no longer work. Has done nothing for four years. At time of examination is anemic; the skin is dry and he is much emaciated. The lower border of the stomach is found below the umbilicus and there may occasionally be seen anti-peristaltic movements and a ballooning of the stomach.

Test examination revealed hydrochloric acid and lactic acid—no Faden bacilli, but a great number of sarcinæ. The diagnosis was made of a benign obstruction at the pylorus and the case was transferred to the surgical clinic, where Dr. Parmenter performed a gastro-enterostomy. The tumor was examined afterward microscopically and it was found to be fibrous tissue. The case died of shock.

From the above I am led to conclude that (1) the Oppler-Boas bacillus is a very valuable aid in the diagnosis of gastric carcinoma.

(2) That it may easily be demonstrated and therefore is of practical importance.

400 FRANKLIN ST.

